# Supplementary material for: Efferocytosis of SARS-CoV-2-infected dying cells impairs macrophage anti-inflammatory functions and clearance of apoptotic cells
Source: eLife. 2022 Jun 6;11:e74443. doi: 10.7554/eLife.74443 (PMC9262386; doi:10.7554/eLife.74443)
Supplement: Supplementary file 1. [file elife-74443-supp1.docx]

| Term | Gene | | | | |
| --- | --- | --- | --- | --- | --- |
|  | GAS6 | SCARB1 | MARCO | MEGF10 | STAB1 |
| Recognition (eat-me receptors) | MERTK | ITGB3 | MFGE8 | MSR1 | STAB2 |
|  | ADGRB1 | ITGAV | AXL | TIMD4 | AGER |
|  | LRP1 | CD36 | TYRO3 | C1QA | SCARF1 |
|  | RAC2 | RHOBTB1 | DNM1L | ABCA1 | ATG7 |
|  | RAC1 | RHOG | ELMO1 | RUBCN | ATG16L1 |
| Engulfment and processing | RHOH | RAB14 | SLC2A1 | Becn1 | GULP |
|  | TREX1 | TYROBP | SLC12A4 | PIK3C3 |  |
|  | RHOBTB2 | DOCK1 | DNASE2 | ATG5 |  |
