# Supplementary material for: Efferocytosis of SARS-CoV-2-infected dying cells impairs macrophage anti-inflammatory functions and clearance of apoptotic cells
Source: eLife. 2022 Jun 6;11:e74443. doi: 10.7554/eLife.74443 (PMC9262386; doi:10.7554/eLife.74443)
Supplement: Supplementary file 2. [file elife-74443-supp2.docx]

| **Table S1** - COVID-19 patients medical characteristics | | | |
| --- | --- | --- | --- |
|  | **Demographics** | | **%** |
| Number | 4 | |  |
| Age | 73.5 ± 7.7 | |  |
| Female |  | | 50 |
|  | **Comorbidities** | |  |
| Hypertension | 2 | | 50 |
| Obesity | 1 | | 25 |
| Diabetes | 2 | | 50 |
| History of smoking | 1 | | 25 |
| Heart disease | 3 | | 75 |
|  | **Laboratorial findings** | |  |
| ALT | 620.25 ± 1106.36 | |  |
| AST | 1234 ± 2278.72 | |  |
| CRP (mg/dL) | 14.37 ± 5.04 | |  |
| D-Dimers (μg/mL) | 2.38 ± 1.45 | |  |
| LDH (mmol/L) | 3.05 ± 1.85 | |  |
| Urea | 147.45 ± 35.68 | |  |
|  | **Respiratory status** | |  |
| Mechanical ventilation | 3 | | 75 |
| Nasal-cannula oxygen | 3 | | 75 |
| pO2 | 72.65 ± 13.53 | |  |
| SatO2 | 90.2 ± 7.2 | |  |
|  | **Disease severity** | |  |
| Mild | 0 | | 0 |
| Moderate | 0 | | 0 |
| Severe | 4 | | 100 |
|  | **Outcome** | |  |
| Death | 4 | | 0 |
|  | |  |  |
|  | |  |  |
| *CRP: C-reactive protein (normal value <0.5 mg/dl); **D-dimers (normal value <0.5 μg/ml); : | | | |
| #LDH: lactate dehydrogenase (normal range: 120–246 U/liter); | | | |
